# Simultaneous Multifocal Paradoxical Embolism in an Elderly Patient with Patent Foramen Ovale: A Case Report

**DOI:** 10.7759/cureus.6992

**Published:** 2020-02-14

**Authors:** Hassan M Lak, Taha Ahmed, Raunak Nair, Anjli Maroo

**Affiliations:** 1 Internal Medicine, Cleveland Clinic - Fairview Hospital, Cleveland, USA; 2 Internal Medicine, Cleveland Clinic Foundation, Cleveland, USA; 3 Cardiology, Cleveland Clinic - Fairview Hospital, Cleveland, USA

**Keywords:** pfo, saddle, embolism, amplatzer

## Abstract

About one-third of ischemic strokes may be associated with a patent foramen ovale (PFO). This article presents an unusual case of a 68-year-old woman with simultaneous paradoxical thrombo-embolization to different systemic sites. The patient presented initially with visual deficits and intracerebellar hemorrhage but was found to have concomitant saddle pulmonary embolism, sub-acute cerebral infarction with focal neurological deficits, and thromboembolism to the superior mesenteric artery (SMA) that resulted in an ischemic bowel. The unifying diagnosis was paradoxical embolism through a PFO and an atrial septal aneurysm with high-risk features. The patient underwent percutaneous closure of the PFO with an Amplatzer device.

## Introduction

Paradoxical embolism (PDE) or crossed embolism refers to an embolus of venous origin which is carried directly into the arterial circulatory system, or vice versa through a right to left intracardiac shunt such as a patent foramen ovale (PFO) [1]. In about 25% of people, the foramen ovale fails to close properly after birth, leaving them with a PFO. PFO is usually asymptomatic; however, it may play a role in the development of a cryptogenic stroke (CS). About one-third of cryptogenic ischemic strokes may be associated with a PFO [2]. Additionally, in patients who have had a cryptogenic stroke, the probability of having a PFO increases to about 40% to 50% [3]. Cerebral circulation is usually the most common site for thrombus dislodgement in PDE, manifesting as an ischemic stroke. Paradoxical embolism has a diverse clinical presentation and the nature and complications of this pathology can be serious and potentially life-threatening, which requires a high index of suspicion for its diagnosis [4]. The initial workup for PDE includes computed tomography (CT) of the head, magnetic resonance imaging (MRI) of the brain, laboratory investigations including D-Dimer, electrocardiogram (ECG), and echocardiography (transthoracic (TTE) and possibly transesophageal (TEE)) [5]. Our case describes a multisystemic presentation of PDE manifesting as simultaneous ischemic stroke, pulmonary embolism, and superior mesenteric artery (SMA) thromboembolism in the clinical context of a PFO [6].

## Case presentation

A 68-year-old female with a past medical history of hypertension was admitted with complaints of acute visual disturbance, manifesting as partial hemianopia without headache. On admission, her vital signs included a blood pressure of 161/110 mm Hg, a regular heart rate of 94 beats/minute, respiratory rate of 20 breaths per minute, and oxygen saturation (SaO_2_) of 93% on room air. She was afebrile and was alert and oriented to time, place and person.

The cardiac examination showed normal S1 and S2 without murmurs, rubs, or gallops, and chest examination showed lungs clear to auscultation bilaterally. There were no signs of deep venous thrombosis (DVT) on bilateral calf examination. Neurological examination showed no weakness or sensory loss. A computed tomography (CT) scan of the brain showed a 7-mm hyperdensity in the left cerebellar area. Tissue plasminogen activator (TPA) was not given due to uncertainty about the time of onset of the cerebrovascular symptoms. The patient was started on aspirin 325 mg and atorvastatin 40 mg daily.

**Figure 1:**
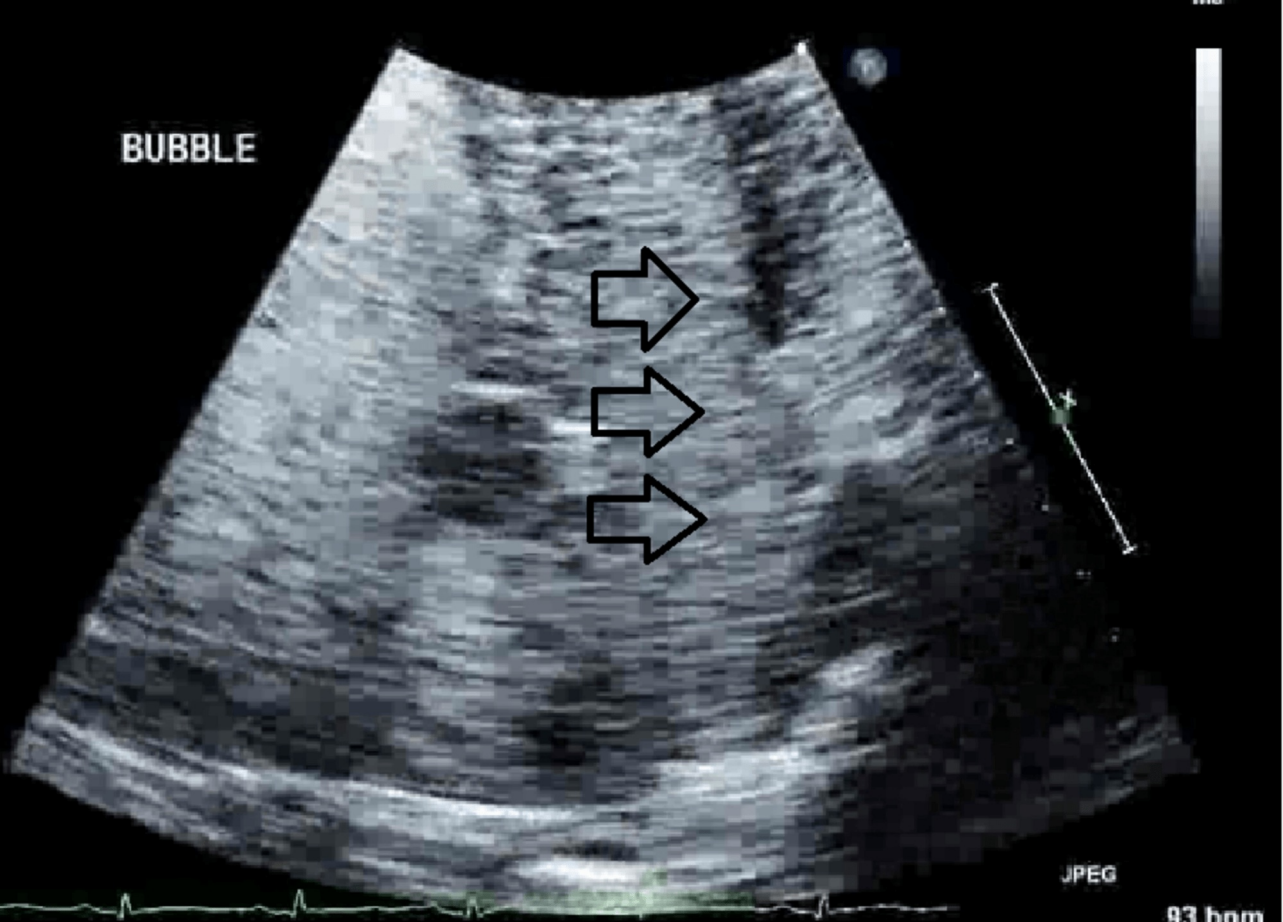
Transthoracic echocardiogram showing complete opacification of the left atrium and left ventricle within 2 cycles of injection of agitated saline contrast (arrows)

The next day, the patient had a sudden onset of hypoxia, with an oxygen saturation of 80%, as well as a left-sided facial droop and left-sided motor weakness. D-dimer was elevated at 17,900 micrograms/liter (normal reference range: 400 mcg/liter). Urgent computed tomography angiography (CTA) of the chest revealed a saddle pulmonary embolus with a moderate to severe degree of clot burden bilaterally and evidence of right heart strain. TTE revealed that the right ventricular systolic function was moderately decreased regionally, and the estimated right ventricular systolic pressure was 43 mm Hg, consistent with mild pulmonary hypertension. Agitated saline contrast exam showed complete opacification of the left atrium and left ventricle within two cardiac cycles, indicative of significant right to left shunting (Figure 1).

**Figure 2:**
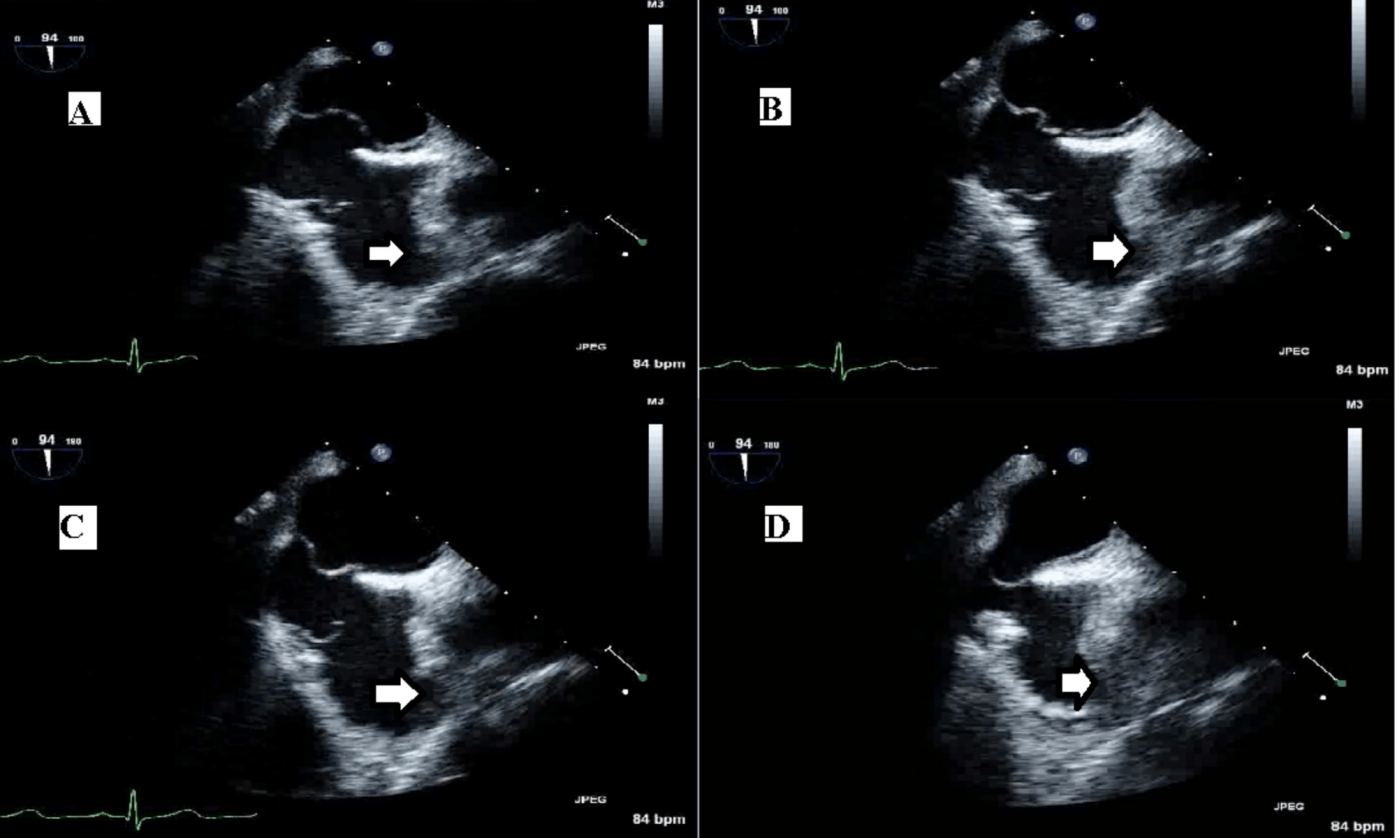
Serial images (A, B, C, D) through transesophageal echocardiogram showing PFO (arrows) and atrial septal aneurysm PFO, patent foramen ovale.

The interatrial septum was mobile but appeared to be structurally intact, with only a small PFO. Subacute infarcts were seen in the right occipital-parietal area posteriorly on CT brain imaging. The patient’s recent cerebrovascular accident and the significant risk of hemorrhagic conversion precluded her from receiving tissue plasminogen activator (TPA). She was anticoagulated with intravenous unfractionated heparin. This was subsequently changed to argatroban due to concern for heparin-induced thrombocytopenia. TEE confirmed PFO and an atrial septal aneurysm with high-risk features: total septal excursion of 19 mm, 10-mm unilateral excursion toward each side in separate views, and 2.6-mm separation between the two layers of the interatrial septum (IAS) (Figure 2).

While recovering from the cerebrovascular accidents and the saddle pulmonary embolism, the patient developed an acute abdomen. CT angiography revealed a nearly occlusive thrombus in the superior mesenteric artery (SMA) that required embolectomy and small bowel resection. Antiplatelet therapy (clopidogrel) was added to her antithrombotic regimen, and she was eventually transitioned to warfarin and clopidogrel.

**Figure 3:**
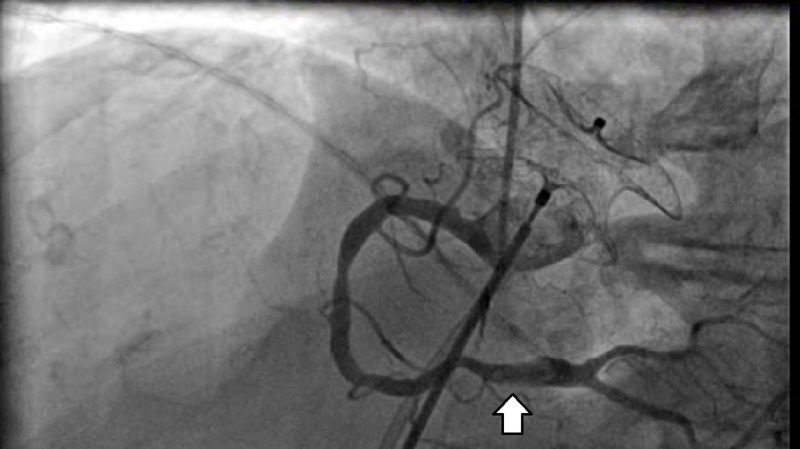
Cardiac catheterization showing occlusive thrombus in the right coronary artery (arrow)

After recovery from her bowel surgery, the patient underwent a planned percutaneous closure of her PFO, using bivalirudin as an anticoagulant. During this procedure, intracardiac ultrasound (ICE) revealed a mobile thrombus in the main pulmonary artery, as well as thrombi in the superior portion of the right atrium and the superior vena cava. Shunting across the PFO was assessed by agitated saline contrast exam and was graded as a severe right to left shunting in the presence of low to normal right heart filling pressures (right atrial mean pressure 5 mm Hg, right ventricular pressure 20/6 mm Hg). During the procedure, the patient developed acute ST-segment elevation inferiorly. Emergent coronary angiography revealed a thrombus in the distal right coronary artery (RCA) (Figure 3).

**Figure 4:**
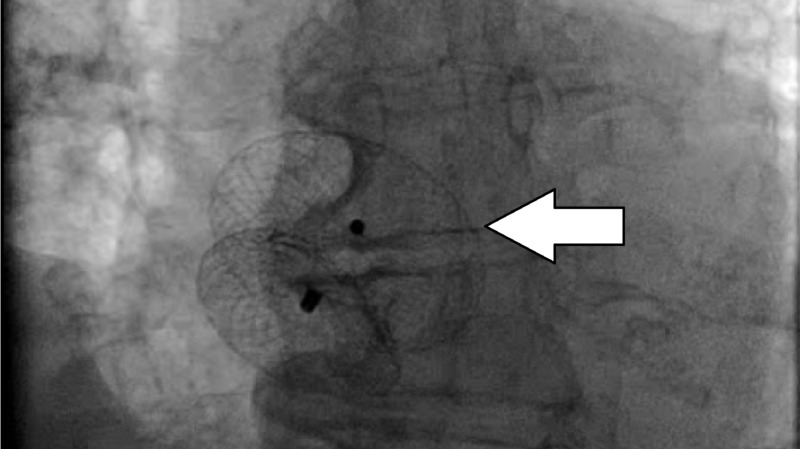
#28 Amplatzer septal occluder (arrow)

**Figure 5:**
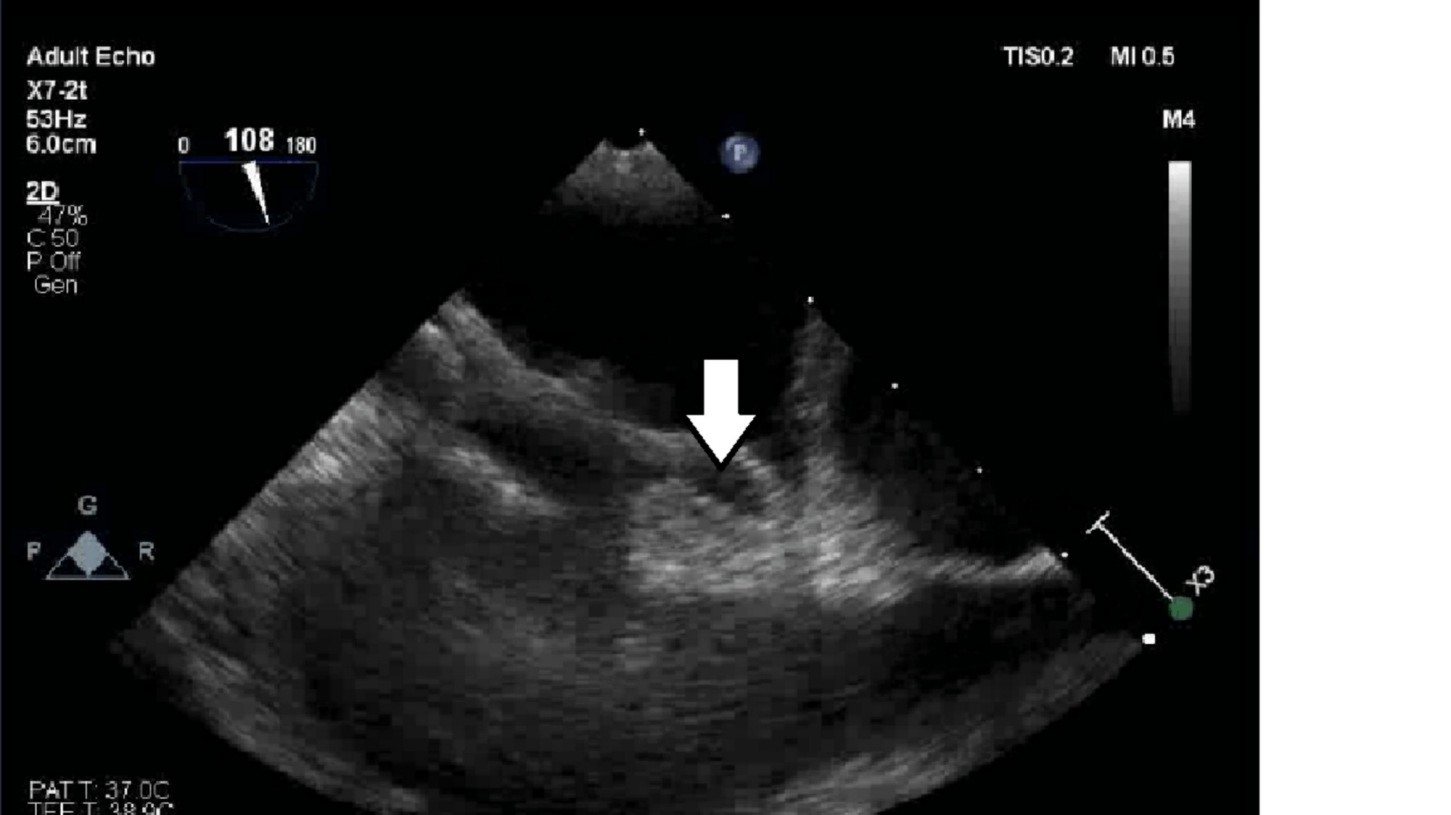
Transesophageal echocardiography showing #28 Amplatzer septal occluder device (arrow)

The patient was loaded with ticagrelor, and cangrelor was given intravenously with tirofiban. The patient underwent aspiration thrombectomy of the RCA, which restored normal coronary flow. Her access sheaths began to develop thrombi, and the anticoagulant was changed to argatroban. The PFO was closed with a #28 Amplatzer septal occluder device (Figures 4-5). The patient stabilized post-procedure, and she was transitioned to warfarin and clopidogrel for 6 months after device implantation.

## Discussion

PDE is defined as an incidental manifestation of dislodgment of a venous thrombus into the systemic circulation via an intracardiac shunt or arteriovenous malformation (AVM) in the lungs. In the vast majority of cases, it presents clinically as an acute or transitory ischemic stroke with a two-fold long-term increase in mortality and disability risk [7]. The possibility of paradoxical embolization should be entertained when no other identifiable source of an embolus (such as atrial fibrillation) can be found. Patients should be screened for any history of previous stroke, DVT, or cardiovascular disease (CVD). Additionally, a careful physical examination to look for signs of congenital heart defects, such as digital clubbing, fixed splitting of S2, or right ventricular hypertrophy, should be performed. Other common causes of stroke should be excluded first since paradoxical embolism is a diagnosis of exclusion. To evaluate the cause of systemic embolization, a TTE with color-flow Doppler, TEE, transcranial Doppler sonography (TCD), and ear oximetry can be employed [8].

The long-term follow-up data from the clinical trials randomizing cryptogenic stroke patients to percutaneous PFO closure or medical therapy have shown that with good patient selection, transcatheter PFO closure significantly reduces the risk of recurrent stroke compared with medical therapy. Also, no increased risk of serious adverse events including major bleeding was found with the prior [9]. A patient-level meta-analysis of the earlier PFO and stroke trials (CLOSURE I, PC, and RESPECT) confirmed the superiority of PFO closure over medical therapy for secondary prevention of stroke [Hazard ratio (HR): 0.58; p = 0.043)] among 2,303 patients with a cryptogenic stroke event. Additionally, the use of the Amplatzer PFO occluder device in the PC and RESPECT trials showed a more robust benefit (HR: 0.39; p = 0.013) [10]. Two newer randomized, multicenter, open-label trials CLOSE (NCT00562289) and Gore REDUCE (NCT00738894) also showed transcatheter PFO closure to be superior to medical therapy alone in patients with cryptogenic stroke. Also, in patients with cryptogenic stroke who have certain echocardiographic features (i.e., the presence of large shunt or atrial septal aneurysm), fewer recurrent strokes were seen from the long-term follow-up of RESPECT and the CLOSE trial [11]. Both CLOSE and REDUCE have stricter inclusion criteria enrolling only the patients whose stroke was secondary to PFO rather than another etiology (such as large artery atherosclerosis, atrial fibrillation, or small vessel disease). All 5 trials have allowed the recognition of a “purer” patient population at a higher risk of recurrent PDE (i.e., prior venous thromboembolism, multifocal cerebral defects). The three high-risk features for the interatrial septum are 20-mm side-to-side excursion, unilateral 10-mm excursion of a mobile IAS, or >2.0-mm separation between the two membranes of the IAS.

The greatest benefit from percutaneous PFO closure can be expected in patients with no other cardiovascular stroke culprits on laboratory analyses/ imaging (e.g. severe valvular pathology or hypercoagulable disorder), no uncontrolled risk factors (e.g. hypertension or diabetes), no atrial fibrillation or flutter, and no poor prognostic markers. However, an increased risk of atrial fibrillation has been found with PFO closure from the systematic review and meta-analysis from these trials [9]. The DEFENSE-PFO trial also showed that PFO closure is beneficial for preventing stroke in those with high-risk features of the IAS [12]. The optimum anticoagulant strategy has been addressed by recent trials. The NAVIGATE ESUS trial showed no significant difference in the risk of recurrent ischemic stroke between rivaroxaban and aspirin (hazard ratio [HR] 0·54; 95% CI 0·22-1·36) among patients with known PFO with no difference in the risks of major bleeding (in patients with PFO detected (HR 2·05; 95% CI 0·51-8·18) and in those without PFO detected (HR 2·82; 95% CI 1·69-4·70; p interaction=0·68) [13]. Similarly, no significant difference was shown by the three RCTs (randomized controlled trials) comparing anticoagulation versus antiplatelet therapy in cryptogenic stroke patients [14].

Embolism to more than one location is a rare phenomenon. According to one study, PDE may complicate two separate embolic sites in 23% of the cases and three different sites in only 10% of the reported cases [15]. Caretta G *et al*. reported a rare phenomenon of acute pulmonary thromboembolism complicated by a multiorgan PDE associated with a PFO. In that case, the patient was initially treated with TPA given her hemodynamic stability and no contraindications to thrombolysis. PFO closure was not performed because long-lasting oral anticoagulation therapy was necessary given the absence of a demonstrated source of systemic emboli [16]. Another study reported by Islam *et al*. [17] described a case of PDE with PFO simultaneously involving four organ systems: pulmonary circulation, cerebrum, left upper extremity, and the coronary artery. The patient was successfully treated with heparin and intravenous t-PA. Another exceedingly rare case is a study reported by Turedi S *et al*. with PDE involving pulmonary, renal, splenic, and hepatic artery. The patient was treated with heparin and was subsequently referred for PFO closure and implantation of an inferior vena cava filter [18].

Our patient represents one of these rare cases of multi-site embolism. She was initially treated with unfractionated heparin. However, due to concern for heparin-induced thrombocytopenia, she was transitioned to argatroban. After she underwent successful closure of PFO, she was started on warfarin to reduce her long-term risk of recurrent pulmonary embolism as well as clopidogrel for 6 months post device implantation. Despite the extensive nature of her embolism, anticoagulation plus definitive therapy of her PFO with percutaneous closure stabilized her situation. 

## Conclusions

We described the diagnosis and management of a severe form of paradoxical embolism with simultaneous multisystemic involvement. Though dual antiplatelet is a reasonable initial medical strategy, we employed percutaneous closure of PFO as a long-term solution to prevent recurrence of stroke, given the recognition of high-risk features of the interatrial septum. 
